# Experimental study of laser scattering protection system for low-speed aircraft

**DOI:** 10.1371/journal.pone.0308979

**Published:** 2024-08-22

**Authors:** Elliott Donghyun Kim, Gisu Park

**Affiliations:** Department of Aerospace Engineering, Korea Advanced Institute of Science and Technology, Daejeon, Republic of Korea; Guangdong University of Petrochemical Technology, CHINA

## Abstract

This study introduces a laser scattering system to protect a low-speed aircraft. Scattering was selected to reduce the laser’s intensity targeting the sensor of an aircraft and simultaneously maintaining the functionality of aircraft optics. Mie scattering, known for effectively decreasing short-wave infrared light, was employed by utilizing water aerosols having a diameter of 1 to 5 μm. Experimental results regarding the decrease of the laser intensity via scattering confirmed that the theoretical and experimental values resulted in a similar decrease rate under static conditions. To validate the theoretical values, the path length, which the laser passing through water aerosols, was changed. To assess the system’s feasibility in flow conditions, a low-speed wind tunnel was employed to generate two flow speeds: 5.5 m/s and 17.6 m/s. Remarkably, the reduction of laser intensity was only affected by the path length, and was somewhat unaffected regardless of flow speed and the uniformity of the flow, only to the path length. In all cases, the initial laser intensity was set to 10 mW. Under static conditions, the intensity dropped to 8.21 mW, showing a decrease of 17.9%. In flow conditions of 5.5 m/s, 17.6 m/s, and in distorted flow, the laser intensity decreased by 18.3%, 18.1%, and 18% respectively. As a preliminary study, these results demonstrate the system’s capability to protect a low-speed aircraft targeted by lasers even under dynamic flow conditions, may suggest a possibility of providing a practical defence solution.

## 1 Introduction

A low-speed aircraft neutralization system using a high-powered laser was developed owing to its precision, rapid operation, and cost-effectiveness [1, 2]. Recent advancements in the aircraft neutralization system have introduced the “soft kill” technique, wherein a high-powered laser focuses on the aircraft’s electronic circuits to neutralize the optical seeker instead of destructively impacting the aircraft [3]. By aiming the electronic circuits, the laser for the soft kill method requires less power to neutralize a targeted aircraft [4], compared to the “hard kill” technique, which applies physical damage to a targeted aircraft while airborne using chemical lasers capable of generating megawatts of intensity [3–5]. Furthermore, the operation time is significantly lower than that of the previous hard kill system. Based on these advantages, high-powered lasers for soft kill methods have mainly been developed for surface-to-air defences targeting a low-speed aircraft [6].

Following the rapid advancement of soft kill techniques, laser defence systems have also been developed by reducing the intensity of the laser. The laser intensity reduction has been achieved through absorption, reflection, and scattering [7–9]. The laser absorption technique incorporates a unique incident laser emitted from an aircraft that generates heat along the laser trajectory, forming an aerodynamic lens capable of absorbing energy from a high-powered laser directed towards the aircraft [7]. The laser-reflection technique use reflection by coating an ablative material on to the surface of an aircraft, capable of absorbing the energy of a high-powered laser before it reaches the target [9].

Nonetheless, the light-absorption-based protection system, which predominantly converts incident light into heat energy [10], can potentially cause aircraft malfunctions, as the system itself could heat up and damage circuits due to the kilowatt power of the laser. Additionally, experiments have revealed that, in the case of reflection systems, coated aircrafts are more vulnerable to damage. This is due to the presence of impurities such as dust particles on the surface of the aircraft, which cause high-powered lasers to initially melt these impurities. This leads to a greater liability for aircraft destruction in coated aircrafts using the reflection system compared to situations without coating materials [11].

Given the difficulty of using absorption and reflection methods for safeguarding aircraft against lasers, this study aims to introduce a novel concept for a defence system utilizing scattering, which can effectively protect a low-speed aircraft targeted by such lasers. In contrast to the absorption method, the scattering method introduced in this study can be achieved simply by adapting particles along the path of the laser, offering an effective approach, as it eliminates the need for additional emitting devices. Furthermore, compared to the reflection method, the scattering method exclusively safeguards the internal sensors, thereby avoiding any concerns of melting the surface particles. Additionally, because the scattering amount of the laser varies with wavelength, even under identical flow conditions and particle sizes, a laser defence system relying on scattering can effectively reduce the intensity of lasers while simultaneously ensuring the functionality of internal circuits, such as seekers [12]. Therefore, by leveraging the properties of scattering, such as particle size, this study presents a methodology that effectively obstructs lasers while allowing the seeker’s laser, operating at a different wavelengths, to penetrate through, thereby preserving its functionality. Hirst et al. [13] conducted a comprehensive investigation into the spatial intensity distribution of light scattered by individual airborne particles, both spherical and nonspherical. Their work demonstrated how laser light scattering can classify particles based on shape and size, offering valuable insights into the scattering behavior of various particle types. Additionally, the research by Miles et al. [14] on Filtered Rayleigh Scattering (FRS) provides a valuable reference for understanding particle scattering in low-speed and high-speed flows. This demonstrates that a scattering system may operate effectively in low-speed conditions.

In line with this concept, this study presents a novel technology for anti-aircraft protection against lasers that target low-speed aircraft. To effectively defend against lasers, Mie scattering was induced to reduce the laser intensity by employing water aerosols to scatter the incident laser and decrease its intensity. The feasibility of this system was evaluated under various flow conditions. The experiment was conducted in a static state with no flow conditions to establish the fundamental principles of the protection system. Additionally in this condition, the path length was changed to validate the calculations of the theoretical values.

To generate flow, a wind tunnel was operated under two different conditions: a low and high rotation conditions, resulting in average speeds of 5.5 m/s and 17.6 m/s, respectively, simulating the flow conditions experienced by a low-speed aircraft. Moreover, to further assess the scattering system, a distorted flow generator was inserted into the test section of the wind tunnel to induce a non-uniform flow. Under non-uniform flow conditions, such as those influenced by atmospheric turbulences, density changes, occurring aero-optical effects which is possible to affect the scattering amount compared to uniform flow conditions [15, 16]. For the test case with a flow speed of 17.6 m/s, the amount of scattering depending on the flow uniformity was compared and discussed.

## 2 Theoretical background of scattering for a protection system

### 2.1 Scattering theory

Scattering refers to the phenomenon in which moving particles or radiation, such as light or sound, are compelled to deviate from a straight trajectory because of localized non-uniformities present in the medium they traverse [17]. Scattering can be broadly classified into three types: Rayleigh, Mie, and Geometric scattering, which is distinguished by a dimensionless parameter *α*. As shown in Eq (1) [18]

$$\begin{array}{c} \alpha =\frac{\pi D_{p}}{\lambda } \end{array}$$ (1)

where the parameter *α* is determined by the ratio between the diameter of the particle (*D*_*p*_) and the wavelength of the incident light (λ). When *α* is less than 1/10, indicating a significantly smaller particle size, Rayleigh scattering takes place [19]. However, if the particle size is much larger than the wavelength of the light, geometric scattering occurs [20]. Mie scattering occurs within the region where the particle size and wavelength of the incident light are comparable [21, 22].

### 2.2 Selection of wavelength

When developing a protective system for low-speed aircraft, it is essential to specify the laser wavelength. This is because the scattering varies according to the wavelength of the emitted laser. Accordingly, this study presents a protective mechanism to safeguard against lasers within the Short-Wave Infrared (SWIR) range, notably at 1.064 *μ*m, a wavelength commonly adopted for high-powered laser systems [23].

**Fig 1:**
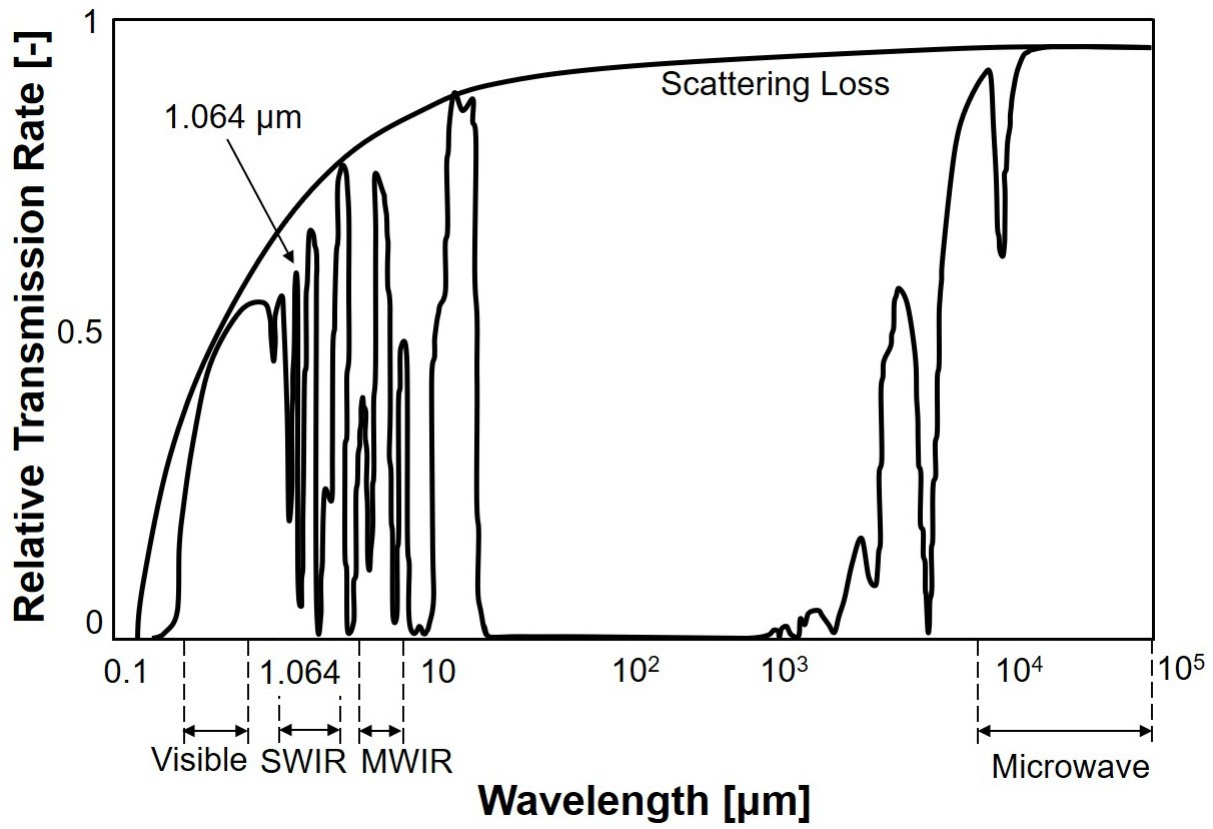
Relative transmission rate by wavelength. Reproduced with permission from [25].

Fig 1 shows the relative transmission rates for various wavelengths under atmospheric conditions. The 1.064 *μ*m wavelength light exhibits a notably superior transmission rate compared to the visible light region [24]. Furthermore, in comparison to higher wavelengths exceeding 10 *μ*m, light with a wavelength of 1.064 *μ*m displays lower scattering losses. As a result, the selection of the 1.064 *μ*m wavelength light for laser systems is driven by its superior transmission rate and reduced scattering losses, setting it apart from other wavelength options. Thus, the scattering system designed for the 1.064 *μ*m wavelength light should employ particles capable of effectively scattering this specific wavelength due to its exceptional transmission properties and minimal scattering losses.

**Fig 2:**
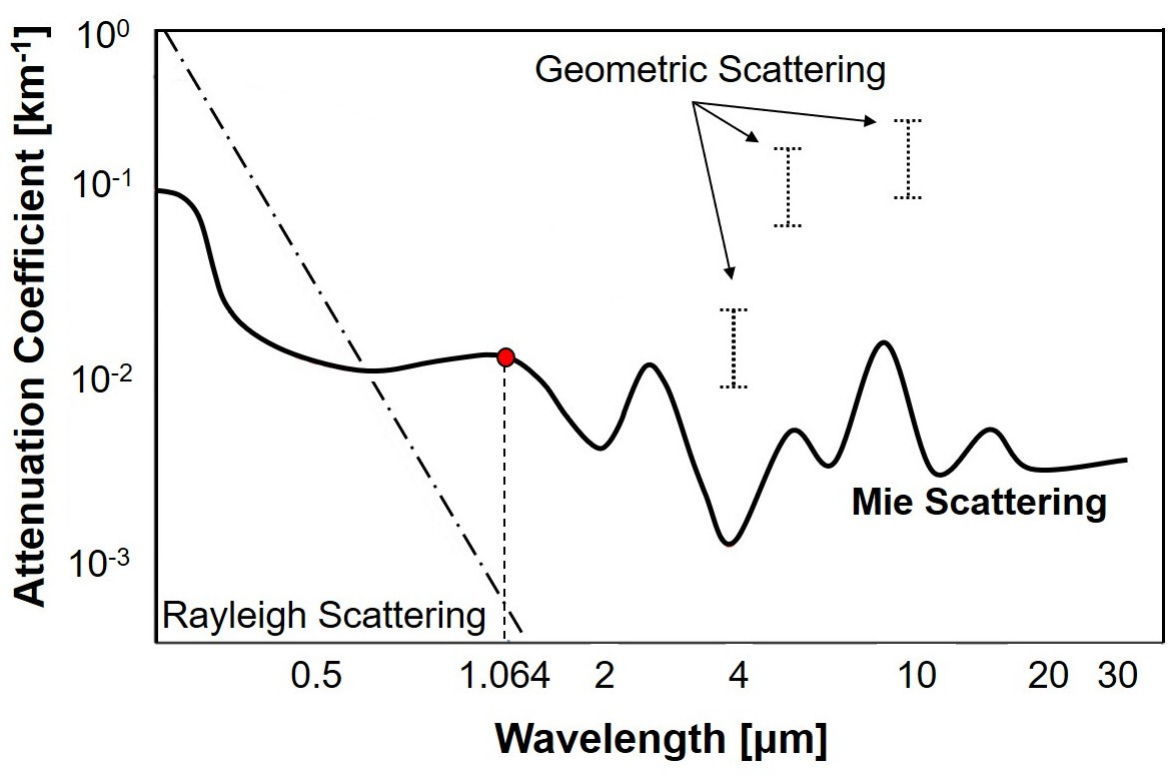
Dominant scattering occurred in different wavelengths. Data adapted from [26].

Fig 2 shows the primary scattering observed at various wavelengths. Specifically, for the 1.064 *μ*m wavelength light, Mie scattering emerges as the dominant mechanism, while Rayleigh scattering exhibits a minimal scattering effect. Furthermore, geometric scattering does not occur within the 1.064 *μ*m wavelength range. As a result, this study primarily focuses on Mie scattering, which is the dominant scattering mechanism within the 1.064 *μ*m wavelength range and serves as the main mechanism for reducing laser intensity.

### 2.3 Selection of scattering material

The effectiveness of Mie scattering is widely recognized when scattering particles exhibit spherical shapes [27]. Therefore, in this study, water aerosols were chosen as the scattering material for the irradiated laser because of the comparatively high surface tension of water. This characteristic enables the possibility of creating a more spherical particles than in other liquids [28].

**Fig 3:**
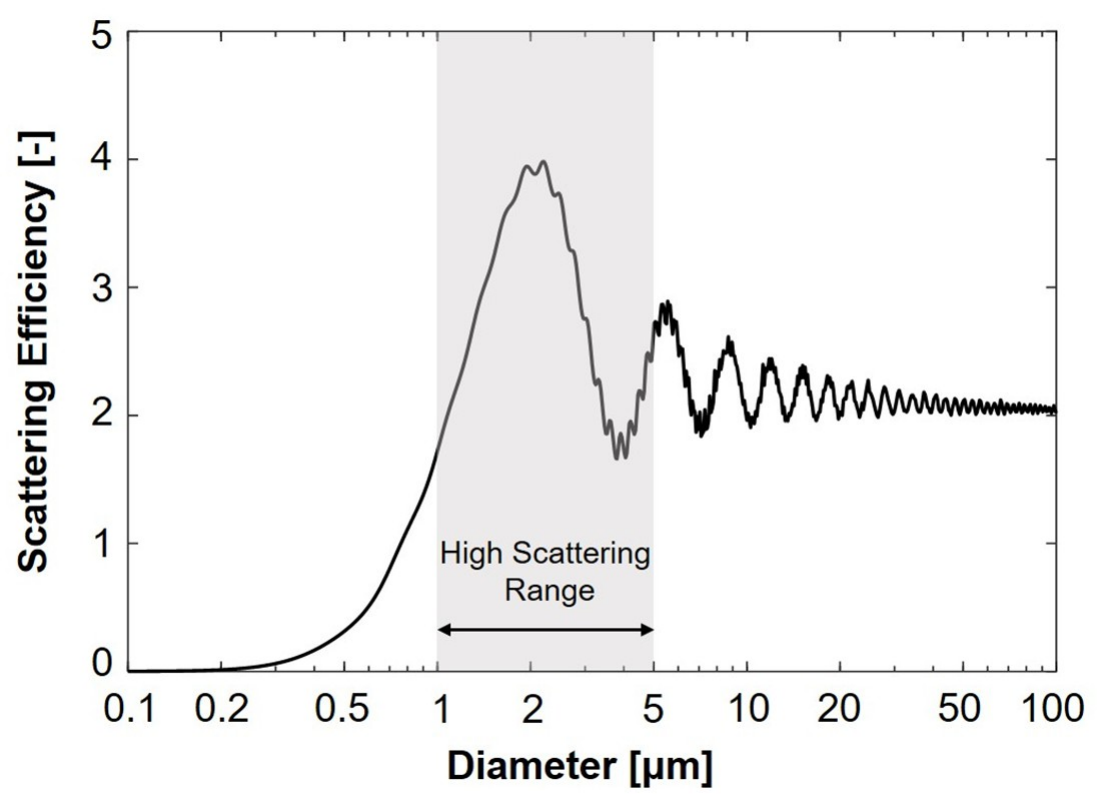
Scattering efficiency by particle diameter.

Fig 3 illustrates the selection of the water aerosol diameter based on the calculation of scattering efficiency in relation to particle size. The MiePlot program [29] released open source by Laven, was employed to analyze water particles scattering 1.064 *μ*m wavelength light across a particle diameter range of 0.1 to 100 *μ*m. The scattering efficiency is defined as the ratio of the power scattered to the incident power on the cross-sectional area of the particles, which is a non-dimensional parameter [30]. The calculation outcomes revealed that particles with diameters ranging from 1 to 5 *μ*m exhibited the highest scattering efficiency, aligning with the Mie scattering regime. Consequently, water aerosol particles with a diameter of 1 to 5 *μ*m, which yield efficient Mie scattering, were chosen as the optimal particle size for the experiment.

### 2.4 Estimated laser intensity

To calculate the reduced intensity of a light due to Mie scattering, a well-established equation known as the “Beer-Lambert law” has been suggested in previous studies [31–33], enabling the calculation of the transmission rate of laser intensity due to absorption. Notably, P. Sprangle et al. [34] modified the Beer-Lambert law specifically for aerosol conditions, to calculate the decreased laser intensity ratio resulting from aerosol scattering, as shown in Eq (2)

$$\begin{array}{c} \frac{I_{initial}}{I_{final}}=\text{exp}\left(-\beta_{A}L\right) \end{array}$$ (2)

where *I*_*initial*_ and *I*_*final*_ represent the intensities of the initial and final lasers, respectively, while *β*_*A*_ and *L* represent the aerosol scattering coefficient and the path length, respectively. The path length is the distance travelled by the laser passing through the water aerosols.

The theoretical aerosol scattering coefficient for a specified wavelength of λ = 1.064 *μ*m was calculated by using the Mie Scattering Calculator [35], which bases its calculations on the equation of scattering for small spherical particles [36]. For input parameters, the sphere diameter was determined as 2.5 *μ*m, as the mean value of the diameters of the selected aerosols ranging from 1 to 5 *μ*m. The refractive index value of 1.33 for the water aerosol 1.33 was used based on Snell’s law [37]. Subsequently, the concentration of a light passing through scattering particles was determined to be 0.042 spheres/*μ*m^3^ representing the average value of aerosol with diameters ranging from 1 to 5 *μ*m. The calculation results reveal that the aerosol scattering coefficient (*β*_*A*_) has a value of 0.015742 mm^−1^. This demonstrates that the Beer-Lambert law for aerosol conditions, Eq (2), is transformed into Eq (3) as presented below

$$\begin{array}{c} \frac{I_{initial}}{I_{final}}=\text{exp}\left(-0.015742\times L\right) \end{array}$$ (3)

indicating that the laser intensity reduction is determined by the path length (*L*), which signifies the distance travelled by the laser through the water aerosols.

### 2.5 Feasibility of the protection system

**Fig 4:**
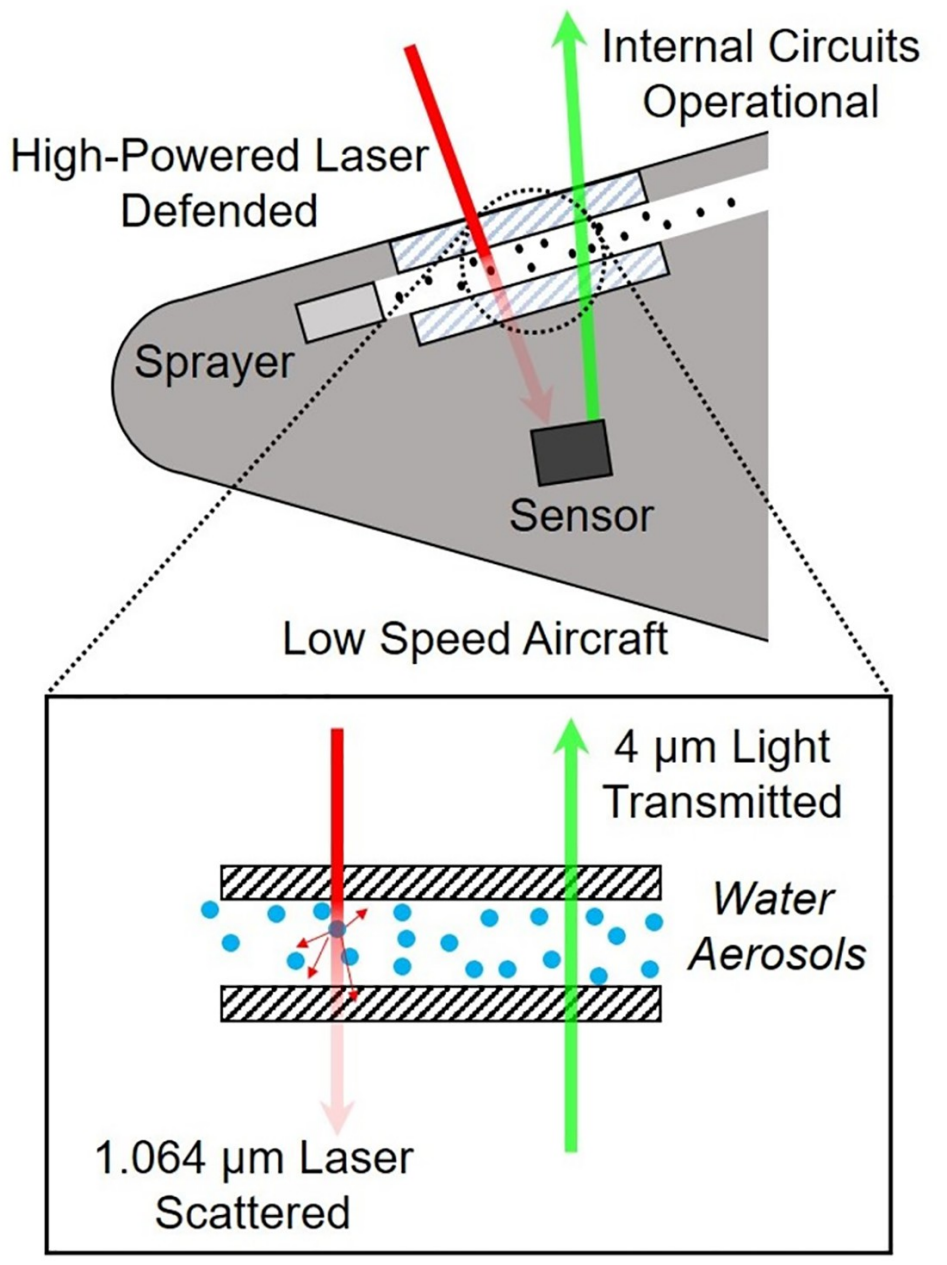
Schematic of a scattering protection system for a low-speed aircraft.

Fig 4 illustrates the application of the protection system. The scattering-based system was designed to be capable of effectively blocking a laser aiming at a low-speed aircraft while simultaneously blocking or affecting other internal sensor components of the aircraft. This concept is possible, due to the fact that the sensors within the aircraft, such as seekers, operating within the mid-wave infrared (MWIR) range and, approximately having a wavelength of 4 to 6 *μ*m [38, 39], exhibit lower scattering efficiency compared to other wavelengths. In addition, scattering was caused by water aerosols in this study, thereby eliminating the occurrence of geometric scattering in the MWIR range. This observation indicates that the utilization of Mie scattering may have the potential to block high-powered lasers with a 1.064 *μ*m wavelength while maintaining the functionality of aircraft seekers. In hypothetical real-world scenarios, when a high-powered laser targets the circuits of a low-speed aircraft, the scattering system reduces the laser’s intensity. Meanwhile, simultaneously, the aircraft’s sensors remain somewhat unaffected, due to their use of different light wavelengths, ensuring the aircraft’s proper functioning.

**Fig 5:**
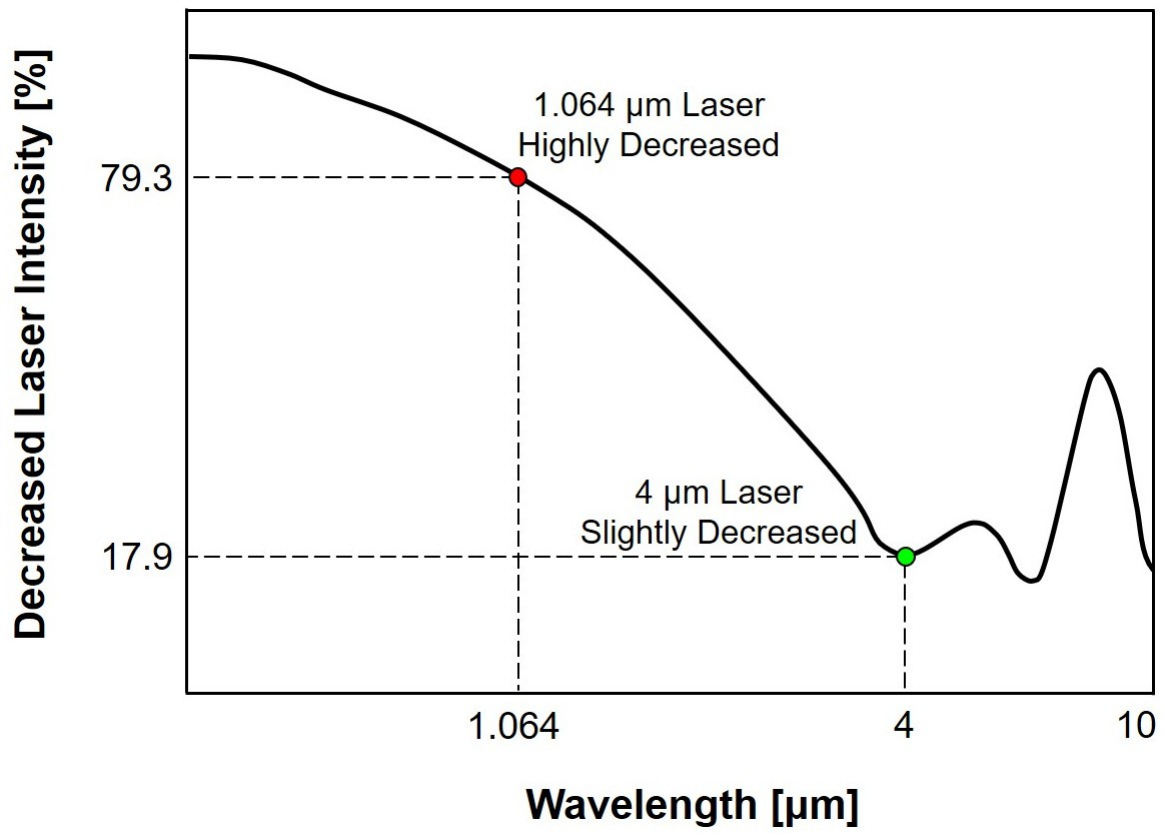
Laser intensity in 100 mm aerosol path length for different wavelengths.

Fig 5 shows the decrease in laser intensity across various wavelengths due to water aerosol scattering with diameters ranging from 1 to 5 *μ*m. The calculations assumed a laser path passing through 100 mm water aerosols. The results indicate that within the 1.064 *μ*m wavelength range, practically employed in high-powered lasers, a reduction of 79.3% is observed. However, within the 4 *μ*m wavelength range, which the aircraft’s internal sensor uses, the decrease is only 17.9%. Thus, an effective low-speed aircraft protection system is introduced in this study.

## 3 Experimental details

### 3.1 Facility and instrumentation

In pursuit of developing a laser protection system to ensure the safety of aircraft, a series of experiments were conducted under static conditions to validate the theoretical values. Furthermore, to simulate the dynamic flow conditions experienced by an aircraft, the experiment involved modifying the flow environment using a wind tunnel, allowing for the practical assessment of laser intensity reduction through water aerosols. Based on the obtained results, a novel protection system was introduced to decrease the intensity of a laser targeting a low-speed aircraft by utilizing water aerosols under flow conditions.

**Fig 6:**
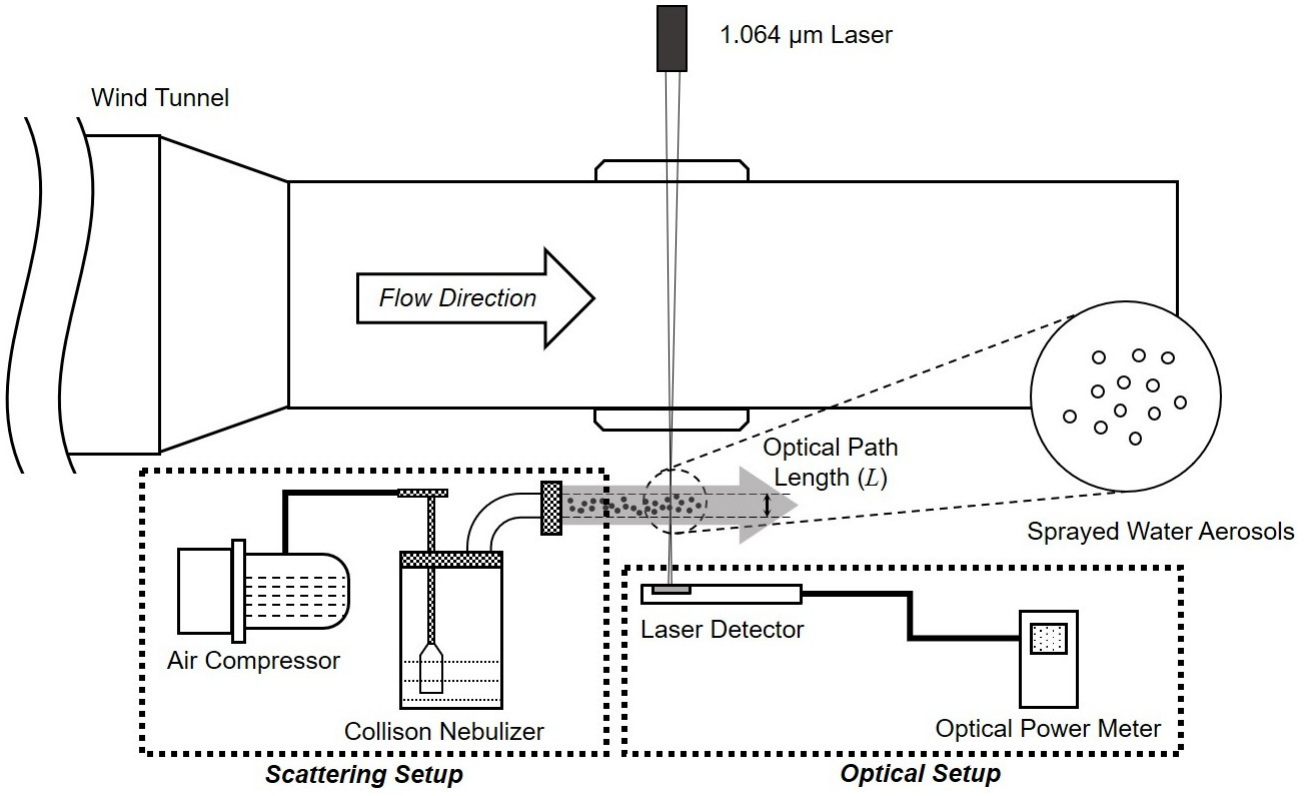
Schematic of the experimental setup to decrease laser intensity by water aerosols under flow conditions.

Fig 6 shows the schematic of the scattering protection system installed in the wind tunnel. The protection system setup consists of two major components: a scattering setup and an optical setup. The scattering setup demonstrates the use of water aerosols to scatter the incident laser, while the optical setup shows the laser and detector employed in the experiment to calculate the reduced laser intensity.

Water aerosols were employed to scatter the laser and reduce its intensity. To induce Mie scattering using water aerosols, a 3-jet type Collison nebulizer (CH Technologies, Collison nebulizer) was utilized in the experiment, as shown in the scattering setup in Fig 6. The Collison nebulizer generates water aerosols with a Mass Median Aerodynamic Diameter (MMAD) of 2.5 *μ*m and a Geometric Standard Deviation (GSD) of 1.8. These values ensured that the diameter of the aerosols fell within the range of 1 *μ*m to 5 *μ*m. The generation of water aerosols using a nebulizer relies on the Bernoulli principle, in which compressed air is introduced at a high velocity through the small orifice of the nebulizer, leading to the fragmentation of water into small droplets. Water aerosol atomization and collision with the wall of the nebulizer result in a further reduction in the droplet size. In addition, gravity causes larger particles to settle on the water surface, whereas specialized traps integrated into a curved outlet tube eliminate additional heavy water particles. In this experiment, an air compressor was used to deliver 20 psi (1.4 bar) to the Collision nebulizer, resulting in a uniform horizontal aerosol flow of 15 mm, ensuring that the 1.064 *μ*m wavelength laser maintained an path length (*L*) of 15 mm in all experimental cases. In addition, a rotational plate was used to support the Collision nebulizer and allowed it to rotate in order to change the path length.

The optical setup in Fig 6 shows the laser and detector used in the experiment. High-powered lasers commonly utilize a wavelength of 1.064 *μ*m. To replicate this particular laser, an Nd: YVO4 laser (CNIL laser, MIL-III-1064 10mW) with a wavelength matching the desired specifications was employed. Although practical lasers that target low-speed aircrafts employ high-powered beams, this experiment utilized a lower-powered laser with the same wavelength, which is suitable for laboratory-scale applications. Furthermore, in the context of scattering, the laser power becomes inconsequential and only the wavelength is significant. Hence, a 1.064 *μ* laser with low power was chosen for this purpose. To detect the 1.064 *μ*m wavelength laser, which is within the infrared range, an optical power detector (Newport, 918D-ST-SL) was employed to capture the laser signal, and a power meter (Newport, 843-R-USB) was utilized to measure the laser intensity.

### 3.2 Flow condition

**Fig 7:**
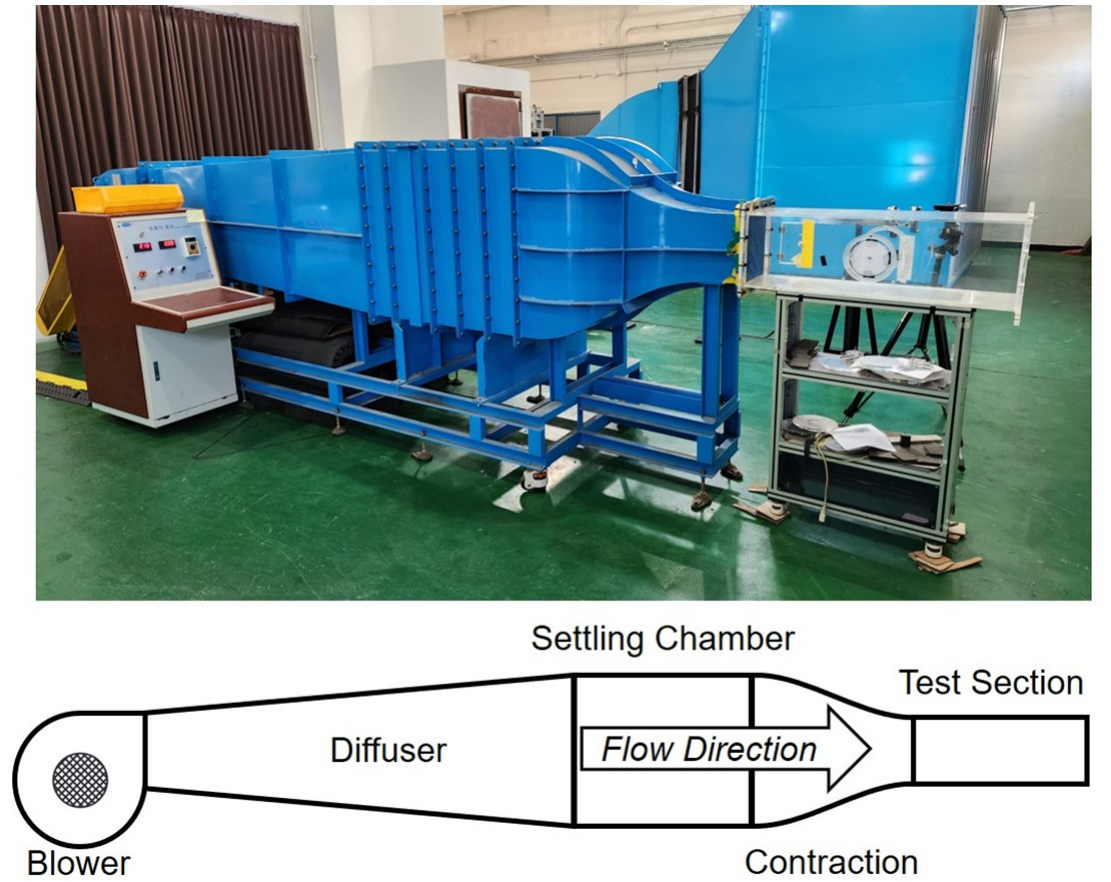
KAIST low-speed wind tunnel and its components.

Fig 7 shows the wind tunnel at Korea Advanced Institute of Science and Technology (KAIST). The wind tunnel [40] contains several key components, including a blower, diffuser, settling chamber, contraction, and a constant-area test section measuring 300 × 300 mm^2^. The blower introduces ambient air into the wind tunnel, whereas the diffuser helps recover the static pressure. The settling chamber straightens the airflow using screens, while the contraction section accelerates the flow from the settling chamber into the test section. The generated flow from the wind tunnel is controlled by adjusting the revolutions per minute (RPM) of the blower’s motor. The wind tunnel utilized in the experiment is a continuous type. The flow is generated by a motor inside the wind tunnel, capable of sustaining continuous testing, thereby allowing the test duration to be set as desired. Similar to the wind tunnel experiments conducted by Mohebbi et al. [41], where a suction open circuit wind tunnel with a test section measuring 100 × 100 cm^2^ was used, the setup used in the experiment also involved detailed aerodynamic testing.

In this experiment, two different flow conditions were used: a low-rotation condition to generate a flow speed of 5.5 m/s and a high-rotation condition for a flow speed of 17.6 m/s. The low-rotation condition of the wind tunnel was obtained by setting the blower’s motor to 10 RPM, generating a flow speed of 5.5 m/s. In contrast, the high-rotation condition was obtained by adjusting the motor to 30 RPM, resulting in a flow speed of 17.6 m/s.

**Fig 8:**
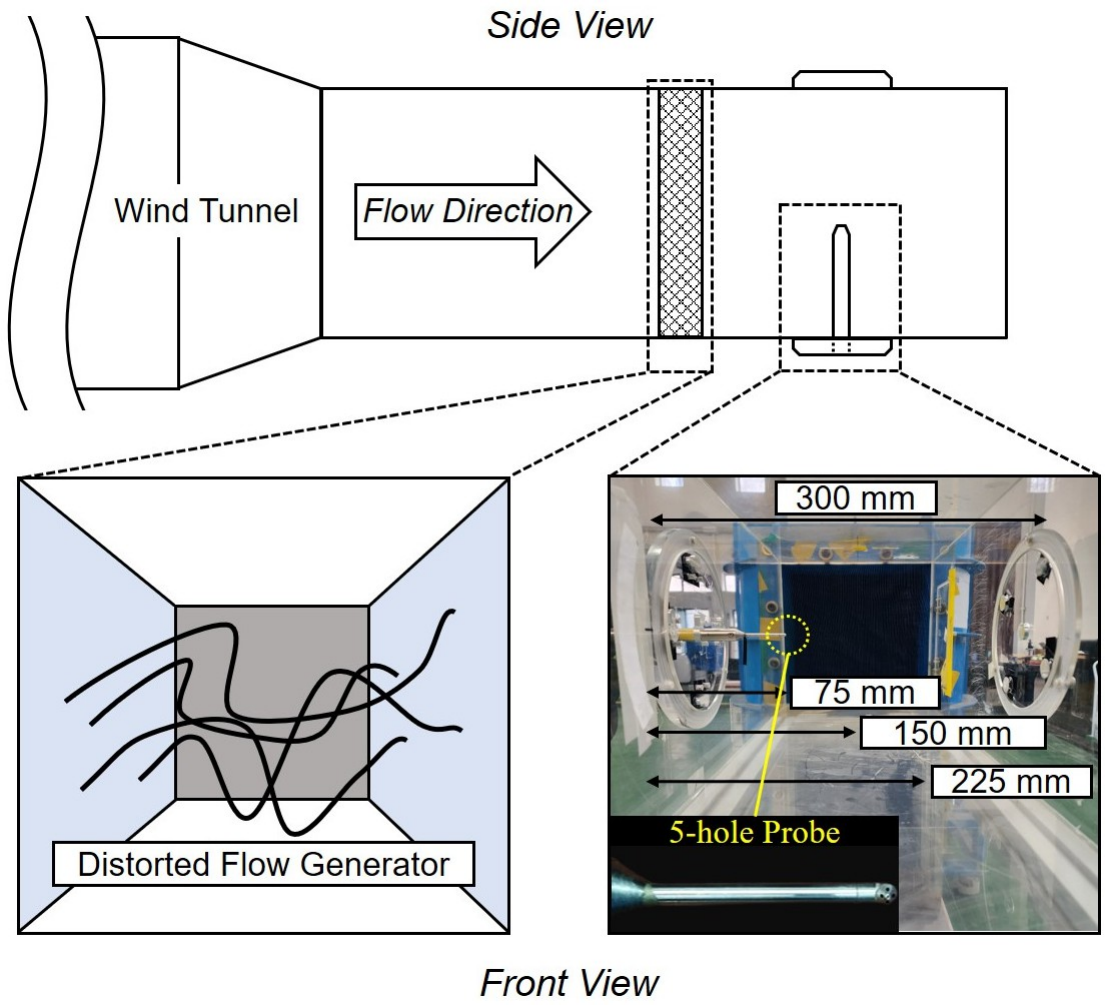
Schematic of the distorted flow generator and a 5-hole probe to measure the flow speed.

Fig 8 shows the experimental setup to simulate the distorted flow conditions that an aircraft may encounter and a 5-hole probe (AeroProbe, Fast Response Probe) used to measure the distortion rate of the flow. This study examines how the laser scattering system is influenced by the non-uniformity of the flow by applying a distorted flow condition for a test case with a flow speed of 17.6 m/s. The distorted flow generator was a rubber coil placed in the test section of the wind tunnel to create a non-uniform flow pattern. The flow deviated from its original direction as it passes through the generator, resulting in a distorted flow. According to Eq (3), the path length is the main factor that reduces the laser intensity under static state conditions.

However, when the flow is distorted, it can alter the interactions among particles in the fluid. This interactions may include increased collisions among particles, impacting the scattering behavior and potentially causing a change in the scattering coefficient (*β*_*A*_). To ascertain the uniformity of the flow, a 5-hole probe was used to measure the distorted flow velocity in the test section. The performance of the distorted flow generator was evaluated by measuring the flow speed at three locations along the test section of the wind tunnel: 75 mm, 150 mm, and 225 mm.

**Fig 9:**
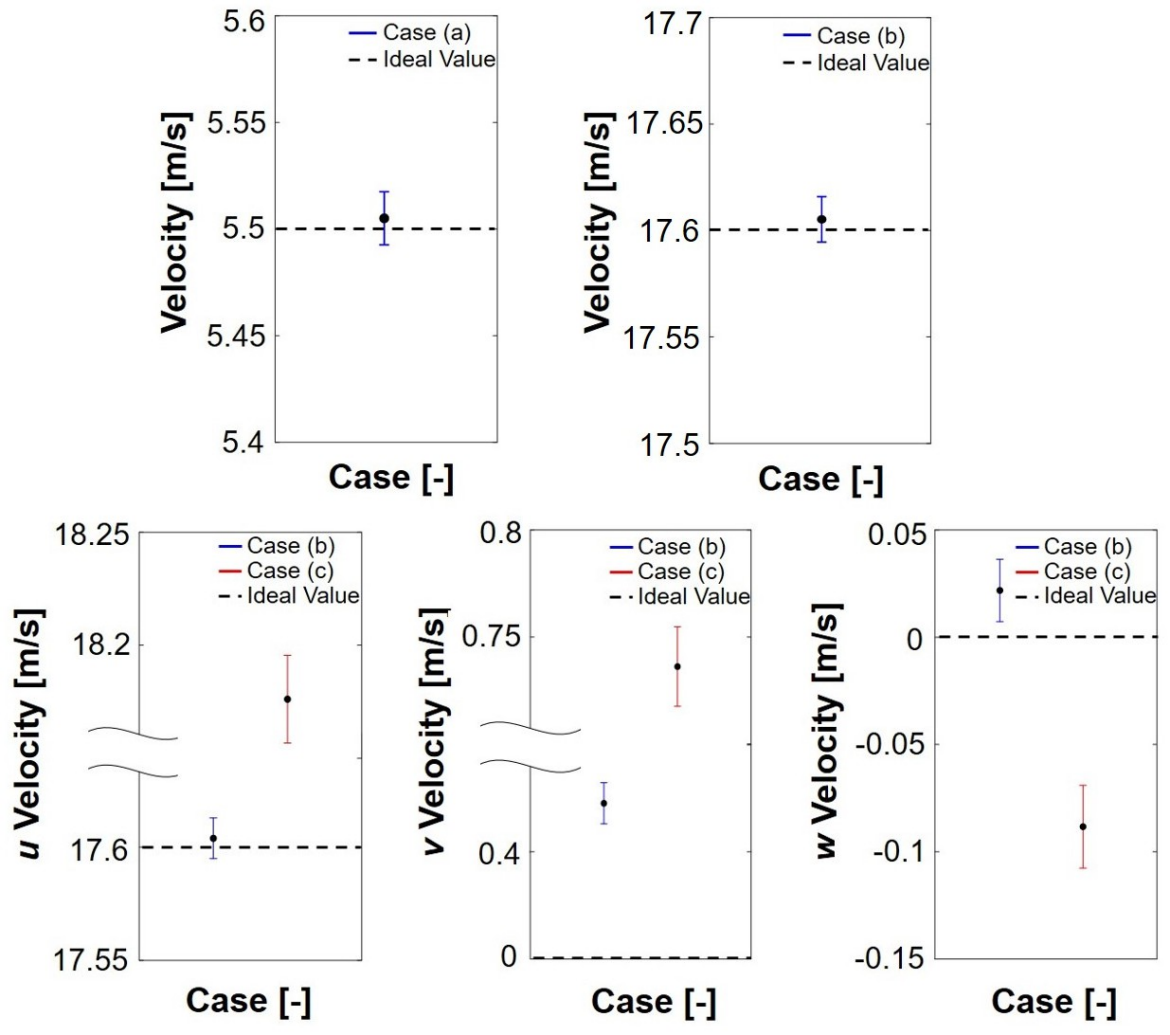
Comparison of flow speeds and the standard deviation rates.

Fig 9 shows the flow velocities under the experimental conditions. The flow cases were classified into three groups: case (a) representing a flow condition of 5.5 m/s; case (b) representing a flow condition of 17.6 m/s; and case (c) representing the distorted flow condition. The results indicate average flow speeds of 5.5 m/s and 17.6 m/s of the wind tunnel conditions, respectively, and are shown with error bars. Moreover, the average and standard deviation of the 17.6 m/s flow speed condition are depicted both with and without the distorted flow generator.

The flow speeds along the *u*, *v*, and *w* axes were determined by employing the 5-hole probe within the test section of the wind tunnel. This enabled a direct comparison of the steady and distorted rates of the generated flow. The comparison between the uniform and distorted flows revealed higher speed values along the *v* and *w* axes, in which the identical values of the speed are 0, indicating the presence of flow distortion. This is because, in an ideal uniform flow along the wind tunnel, the flow speed is only along the u-axis. However, a distorted flow has speed components along the v and w axes, indicating that the flow is not uniformly horizontal, resulting in a distorted flow.

The results reveal that the distorted flow displays a notably higher standard deviation rate of 0.2980 compared to the flow without the distorted flow generator, which registers a standard deviation rate of 0.0342. This emphasizes the increased non-uniformity in the flow field.

## 4 Results and discussion

### 4.1 Theory verification experiment

#### 4.1.1 Static state experiment

To provide validation of the laser scattering system, measurements of the reduced laser intensity were conducted under static conditions. Across all experimental conditions, the total test time was 45 s, and the aerosol spray time was set to 30 s. The stabilization of the laser was maintained for 5 s before the water aerosols were sprayed. The path length for the 1.064 *μ*m wavelength laser passing through the water aerosol was fixed at 15 mm, and the initial laser intensity was set at 10 mW.

**Fig 10:**
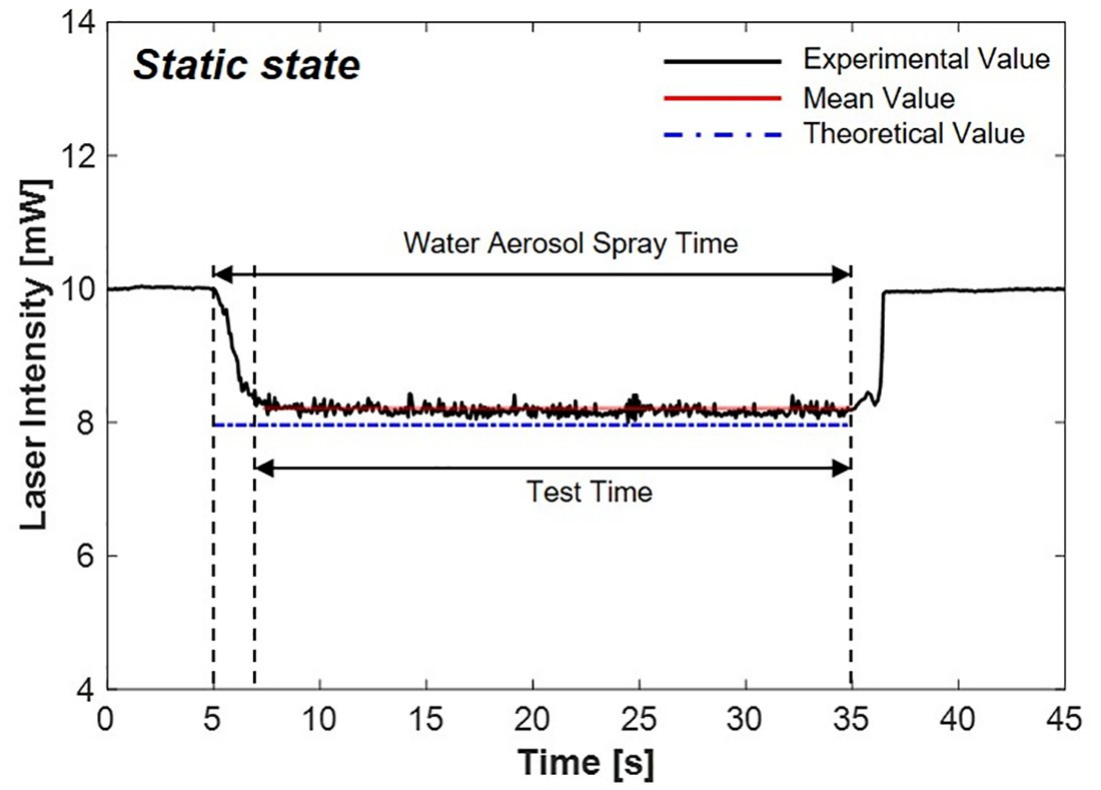
Laser intensity variation by scattering via water aerosols in a static state.

Fig 10 shows the decreased laser intensity under static state conditions. When the 1.064 *μ*m laser encountered the aerosol flow with a path width of 15 mm in the static state, the intensity decreased from 10 mW to 8.21 mW, indicating a 17.9% reduction. The theoretical calculations based on Eq (3) indicate that the laser intensity decreases to 7.89 mW, indicating an error rate of 4.05%. The results demonstrated that under static conditions, the scattering protection system effectively reduced the intensity of the incident laser. This outcome also confirms the accuracy of the calculations used to derive Eq (3).

#### 4.1.2 Static state experiment with rotation

The static state experiment demonstrated the accuracy of the theoretical and experimental decrease in laser intensity, as shown in Eq (3). Nevertheless, Eq (3) indicates that the reduction in laser intensity is solely influenced by the path length. To further validate Eq (3), a rotation experiment was conducted to measure the decrease in laser intensity by water aerosol scattering at different path lengths. Experiments were conducted by rotating the Collison nebulizer to change the path length and observe the resulting difference in laser intensity. The angles were set at 0, 15, 30, 45, 60, and 75 degrees, corresponding to cases 1 through 6, respectively. The rotational experiments were conducted in a static state, where no flow exists, eliminating all variables except the path length. The initial laser intensity was set to 10 mW with a test time of 45 s, and the aerosols were sprayed for 35 s while the Collison nebulizer was rotated. Additionally, in order to mitigate any errors occurring by the change of the path length (*L*), which is the parameter L in Eq (3), the laser alignment was precisely maintained.

**Fig 11:**
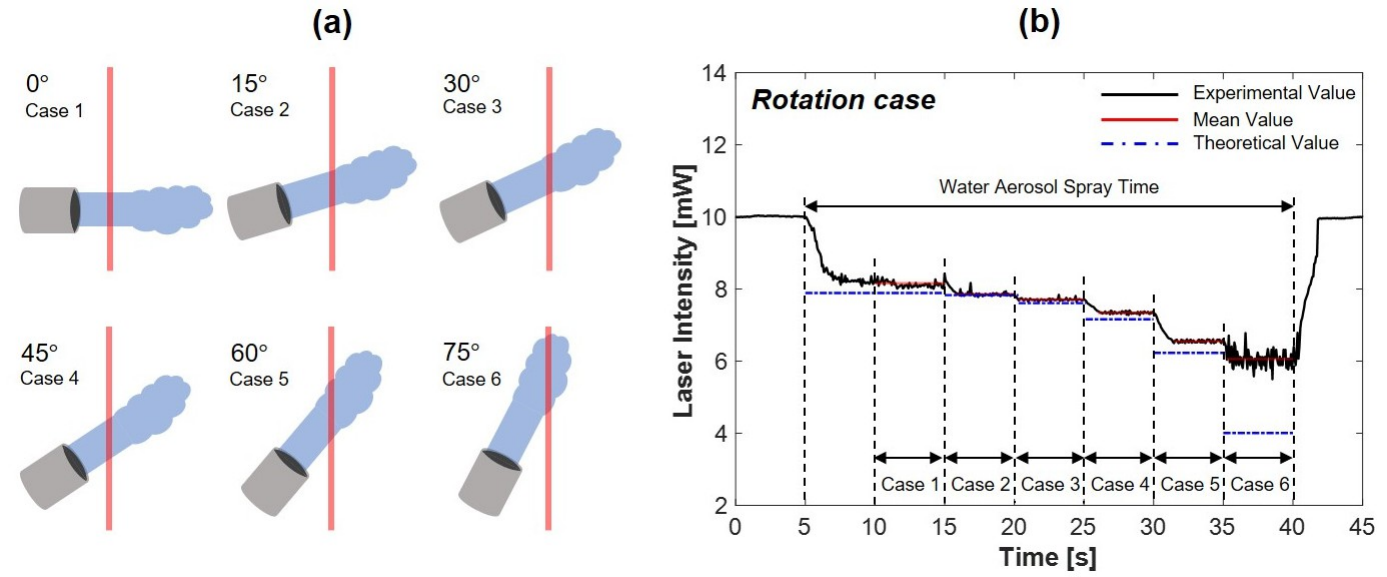
Experiment of rotating the Collison nebulizer (a) schematic of the Collison nebulizer rotation changing the path length; (b) results of the laser intensity change.

Fig 11 shows the schematic and results of the rotational case experiment. The rotation angle was converted into the path length. In case 6, with a rotation angle of 75 degrees, the laser encountered turbulent flow from the sprayed aerosol, resulting in a non-ideal path length and a notably high error rate. However, the results of the remaining tests indicated that the theoretical calculations based on Eq (3) closely align with the experimental values, with an error rate below 6% across all cases.

Table 1 shows the values of the rotation angle of the Collison nebulizer converted into the path length and compares the theoretical and experimental values. The experimental results demonstrated that the calculated values for reduced laser intensity correlated with the experimental values, confirming the feasibility of the protection system. Moreover, this result validates the accuracy of the calculations used to obtain Eq (3).

**Table 1:** Results obtained following a decrease in laser intensity by rotation of the Collison nebulizer.

Rotation Angle [°]	Optical Path Length [mm]	Theoretical Value [mW]	Experimental Value [mW]	Error Rate [%]
0	15.0	7.89	8.16	3.42
15	15.5	7.83	7.86	0.38
30	17.3	7.61	7.71	1.31
45	21.2	7.16	7.34	2.51
60	30.0	6.23	6.55	5.13
75	57.9	4.01	6.06	51.1

### 4.2 Experiment with flow

To demonstrate the system’s capability to operate effectively in flow conditions, experiments involved in flows at different speed, as well as uniform and distorted flow conditions were conducted. In the wind tunnel experiment, the variables remained consistent with those in the static state and the rotation experiment.

#### 4.2.1 Flow speed 5.5 m/s-case (a)

**Fig 12:**
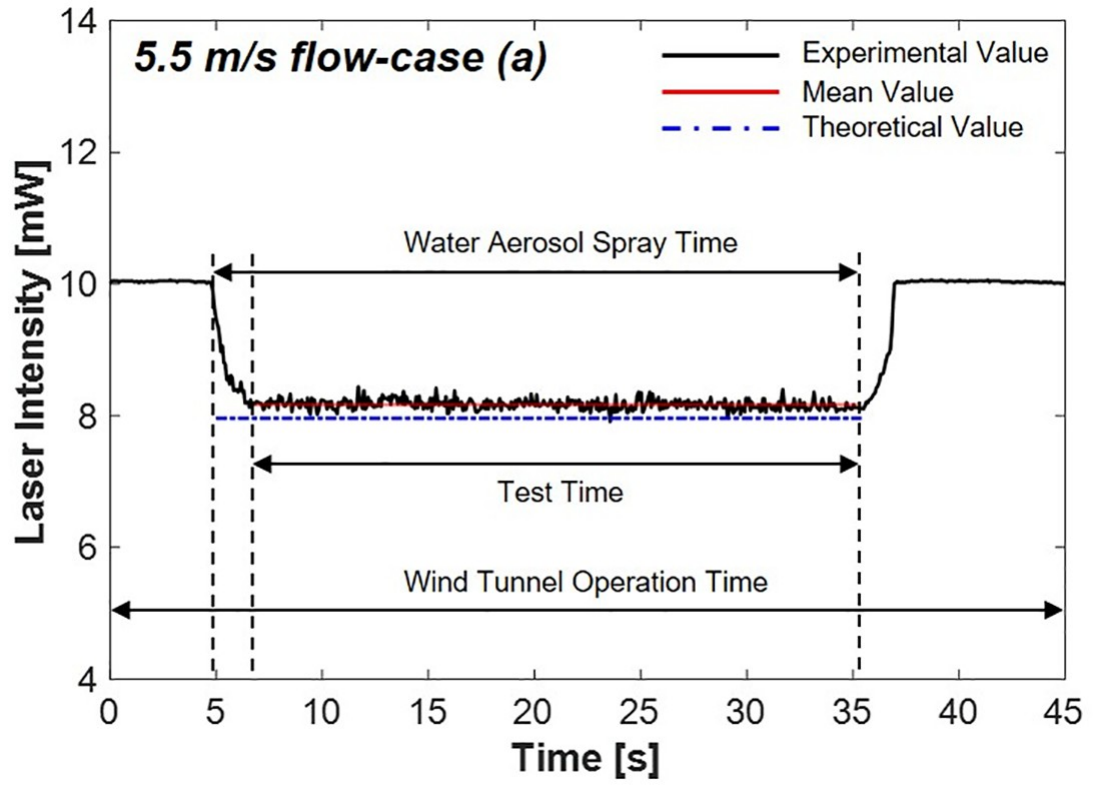
Laser intensity variation by scattering via water aerosols at 5.5 m/s flow speed.

Fig 12 shows the decreased laser intensity caused by water aerosol in the 5.5 m/s flow condition, which is case (a). The experimental setup and procedures were identical to those of the static state experiment, with the wind tunnel running continuously at a flow speed of 5.5 m/s throughout the experiment. The results showed a reduction in the laser intensity to 8.17 mW, indicating an 18.3% decrease, corresponding to an error rate of 3.42% compared with the theoretical value. The results indicate that even under 5.5 m/s flow condition, the scattering system reduces the laser intensity, which is consistent with the results observed in the static state condition experiment.

#### 4.2.2 Flow speed 17.6 m/s-case (b)

**Fig 13:**
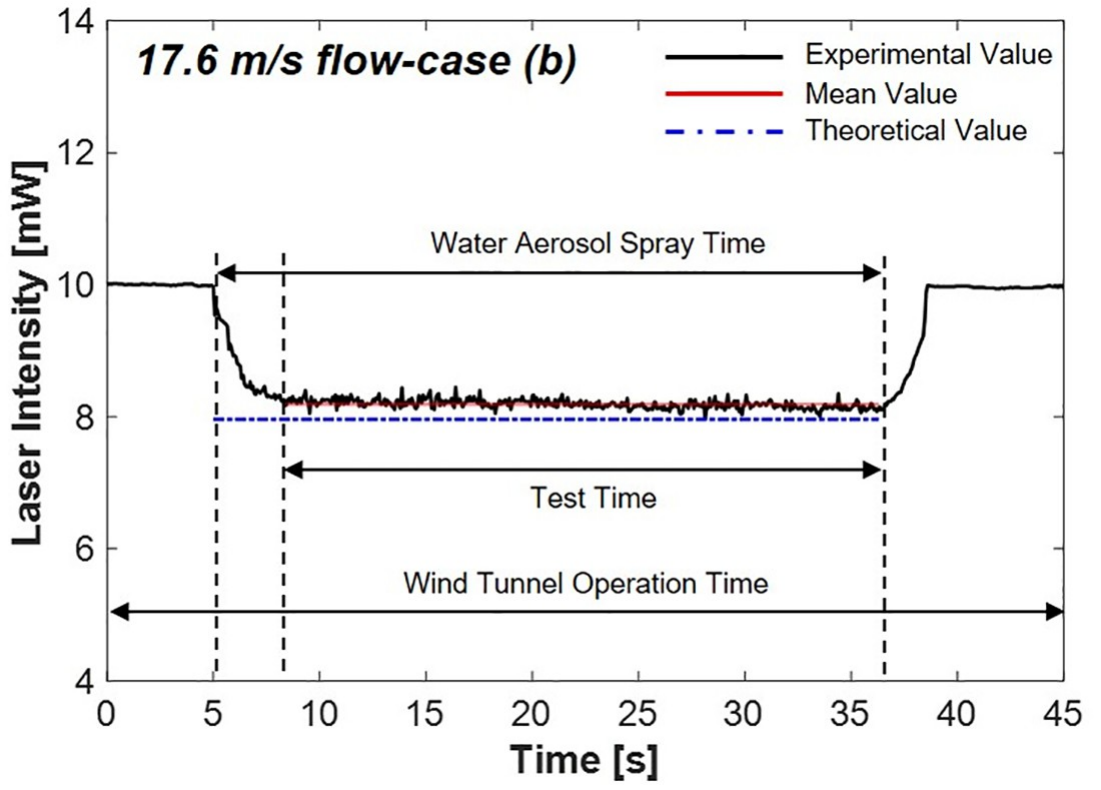
Laser intensity variation by scattering via water aerosols in 17.6 m/s flow speed.

Fig 13 shows the results of the decreased laser intensity in the 17.6 m/s flow condition. The laser intensity decreased to 8.19 mW, exhibiting an error rate of 3.67% compared with the theoretical value. These results are consistent with the findings obtained in the static state and 5.5 m/s flow speed conditions.

Based on the values calculated using Eq (3), the decrease in the laser intensity due to scattering was determined by the path length (*L*). Consequently, it was concluded that the reduction in the laser intensity caused by water aerosols remained unaffected by the flow speed.

#### 4.2.3 Distorted flow-case (c)

**Fig 14:**
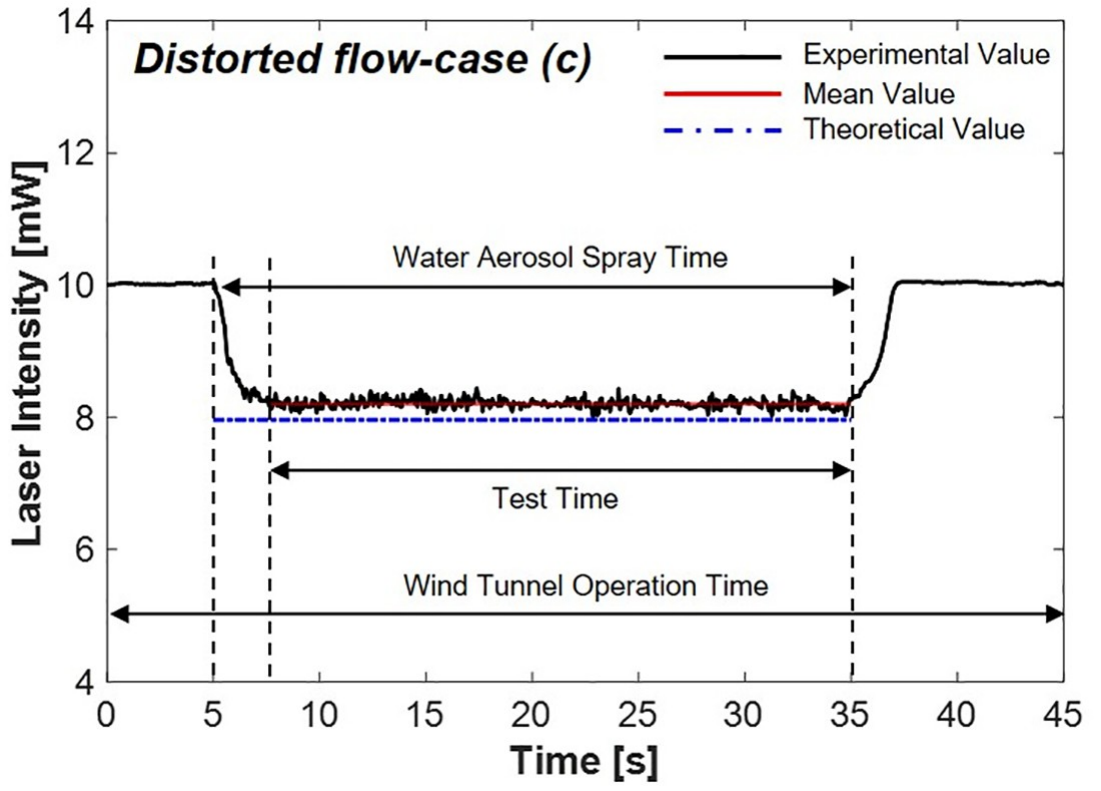
Laser intensity variation by scattering via water aerosols in distorted flow condition.

Fig 14 shows the change in laser intensity by scattering under distorted flow conditions achieved by inserting a distorted flow generator in the test section of the wind tunnel for the 17.6 m/s condition, which corresponds to case (c). The results indicated that a decrease in the laser intensity to 8.20 mW, resulting in an 18% reduction. The error rate was 3.80% compared to the theoretical value. Notably, the outcomes closely resemble those obtained in Fig 13, which is case (b), where the flow speed remains the same, but the flow is steady.

Table 2 summarizes the experimental results. The results demonstrate that under distorted flow conditions, the reduced laser intensity due to water aerosols remained nearly consistent with the outcomes of previous experiments. This emphasizes that the non-uniformity of the distorted flow does not significantly impact the scattering coefficient calculation, as described in Eq (3), which shows that the sole variable affecting the change in laser intensity is the path length. Furthermore, as shown in Fig 1, the high transmittance rate in the atmosphere of the 1.064 *μ*m wavelength laser shows that the uniformity of the flow does not influence the laser’s intensity change.

**Table 2:** Results of decrease laser intensity by each cases.

Experiment Case	Flow Speed [m/s]	Theoretical Value [mW]	Experimental Value [mW]	Error Rate [%]
Static state	0	7.89	8.21	4.05
Case (a)	5.5		8.17	3.42
Case (b)	17.6		8.19	3.67
Case (c)	17.6		8.20	3.80

## 5 Conclusions

In this study, a laser protection system concept was introduced. Scattering was selected as a new defence method because of its ability to protect an aircraft from lasers while ensuring the functionality of aircraft optics when employed under real-flight conditions. Specifically, Mie scattering was used to reduce the intensity of the laser, known to effectively decrease the power of a laser having a wavelength of 1.064 *μ*m used in actual high-powered lasers. Following the principles of small particle theory, water aerosols with a diameter ranging from 1 to 5 *μ*m were selected as the scattering particles to efficiently induce Mie scattering and thereby reduce the intensity of the incident laser.

Confirming the feasibility of the scattering protection system involved conducting a laser intensity decrease experiment in a static state. The experiment showed a decrease in laser intensity from 10 mW to 8.21 mW, indicating the potential for decreasing the 1.064 *μ*m laser intensity through scattering via water aerosols. In addition, the experimental results revealed a notable resemblance to the calculated theoretical values. To validate the accuracy of the theoretical calculations, rotational experiments were conducted, involving variations in the path length, indicating the trajectory of the laser crossing through the water aerosols. As the path length increased, the scattered laser amount also increased, leading to a greater reduction in laser intensity. This outcome affirmed the accuracy of calculations based on the modified Beer-Lambert law, which asserts that the path length is the sole variable of the laser intensity decrease. Moreover, an error rate of less than 6% was shown in each case when the path lengths were adjusted, leading to the conclusion that the calculation results were accurate.

Additionally, a wind tunnel was used to validate the capability of the system to operate under flow conditions. This was based on the consideration that in real-state conditions, an aircraft targeted by a high-powered laser experiences flow. Furthermore, a distorted flow generator was introduced into the tunnel to intentionally create a distorted flow and assess the resulting reduction in laser intensity. The experiments conducted at flow speeds of 5.5 m/s and 17.6 m/s showed values of 8.17 mW and 8.19 mW, respectively, indicating minimal variation. Furthermore, under distorted flow conditions, the degree of flow uniformity did not significantly impact the reduction in laser intensity. In the case of distorted flow in the 17.6 m/s flow case, the laser intensity decreased from 10 mW to 8.20 mW, showing results closely aligned with the previous experiments. Additionally, under both static state and flow conditions, the experimental values exhibited an error rate of less than 5% compared to the calculated values across all cases.

The static state and flow speeds of 5.5 m/s, 17.6 m/s, as well as distorted flow conditions, all exhibit a similar decrease in laser intensity. These results align with the calculations, which indicates that the parameter influencing the change in laser intensity is the path length (*L*). This demonstrates that even in different flow conditions the path length remains constant, leading to nearly identical reductions in laser intensity in each case.

These results indicate that the reduction in laser intensity through water aerosol spraying can be employed in diverse environments, at various air speeds, as well as under distorted flow conditions.

This study is a fundamental research focused on the novel laser scattering protection system concept that concerns the interaction between water aerosol and the laser. Several factors are good to be addressed in the future, such as the effects of vibrations, the challenges of miniaturizing the system for practical implementation and the effect from high-powered laser that is often the case in real scenarios. These aspects seem critical for developing a robust, realistic, and reliable protection system. Future research could address these critical areas to contribute to the design and development of a more effective and practical laser protection system.
